# Value of Online Videos as a Shoulder Injection Training Tool for Physicians and Usability of Current Video Evaluation Tools

**DOI:** 10.3390/ijerph192215177

**Published:** 2022-11-17

**Authors:** Chan Woong Jang, Myeonghwan Bang, Jung Hyun Park, Han Eol Cho

**Affiliations:** 1 Department of Rehabilitation Medicine, Gangnam Severance Hospital, Yonsei University College of Medicine, Seoul 06229, Republic of Korea; 2 Yonsei Graduate School, Department of Integrated Medicine, Yonsei University College of Medicine, Seoul 06229, Republic of Korea; 3 Department of Medical Device Engineering and Management, Yonsei University College of Medicine, Seoul 06229, Republic of Korea; 4 Rehabilitation Institute of Neuromuscular Disease, Yonsei University College of Medicine, Seoul 06229, Republic of Korea; 5 Pulmonary Rehabilitation Center, Gangnam Severance Hospital, Yonsei University College of Medicine, Seoul 06229, Republic of Korea

**Keywords:** education, health personnel, injections, shoulder, social media

## Abstract

This study aimed to evaluate the reliability, overall quality, and educational value of online videos for learning the techniques related to shoulder injection treatments and analyzing the usability of video evaluation tools for musculoskeletal injections. Online video searches were performed in February 2022 using the terms “shoulder injection”, “glenohumeral joint injection”, “acromioclavicular joint injection”, and “subacromial bursa injection.” Included videos were scored by modified DISCERN (mDISCERN), global quality score (GQS), and shoulder injection score (SIS). Correlations between scoring systems were analyzed. Of the 150 videos, 49 (32.67%) contained highly reliable information. Regarding the assessment of overall quality by the GQS, 109 (72.67%) videos were of low quality. Regarding SIS, 114 (76.00%) scored not >5, of which 77 (51.33%) scored <3. Most of the SIS domains were fully explained in <40% of the included videos. A weak positive relationship was noted between the mDISCERN and SIS (r^2^ = 0.38), while a moderately positive relationship was observed between the GQS and SIS (r^2^ = 0.49). The majority of online videos about shoulder injection treatment showed low reliability, overall quality, and educational value. Additionally, a new scoring system is required to accurately evaluate musculoskeletal injection videos for educational purposes.

## 1. Introduction

Shoulder pain is one of the most prevalent problems seen in primary care. Shoulder disorders are considered the third-most common cause of musculoskeletal problems and affect approximately one quarter of the worldwide population [1]. These disorders can cause severe morbidity and reduce a person’s ability to work and perform routine daily activities [2]. There are various causes of shoulder pain, and rotator cuff syndrome, adhesive capsulitis, and subacromial impingement syndrome are the most common problems that cause this pain. Shoulder injection is an essential treatment to improve symptoms and prevent the deterioration of shoulder diseases [1,3].

However, physicians have reported low confidence in performing injection treatments owing to inadequate training [4,5]. Joint injection techniques are conventionally taught by technique demonstration and subsequent supervision of the technique with actual patients, cadavers, or mannequins. The issue is that not all physicians have access to these educational opportunities. One study reported that <50% of primary care physicians received demonstration of this technique [4].

Recently, online videos have emerged as a good alternative for physicians to learn clinical skills with the advancement of the internet [6]. The internet has become the largest up-to-date reservoir of medical information [7]. By taking advantage of its being free and easy to access, online non-face-to-face learning using the data from the internet has become an increasingly attractive method of medical education, especially after the COVID-19 pandemic [8,9]. Recently, medical professionals and students have increasingly used online videos found through YouTube and Google searches to learn medical knowledge [10,11].

However, previous studies evaluating the quality of medical information on social media have mainly focused on medical videos for patients [12]. There were a few reports that studied the suitability of online videos for medical professional education, but the results were inconsistent [10,13,14]. They also did not analyze the practical educational suitability of videos to learn specific procedures. It is unclear, in other words, whether the contents provided in social media videos, which instruct medical professionals about clinical procedures, would be sufficient to offer them accurate and comprehensive information.

Although some studies that have evaluated online videos have developed novel evaluation tools [15], a majority of studies have evaluated the videos with pre-existing tools, such as the modified DISCERN (mDISCERN) and the global quality score (GQS) [15]. Given the fact that these tools were designed with patients in consideration, it is necessary to evaluate whether they are useful for evaluating videos for medical professional education [16,17,18].

Therefore, we tried to evaluate the reliability, overall quality, and educational comprehensiveness of online videos on YouTube and Google, with the goals of educating medical professionals on shoulder injections and identifying whether the current evaluation tools for online videos are appropriate for shoulder injection techniques for medical professionals [16,17,18].

## 2. Materials and Methods

### 2.1. Video Selection Strategy

We selected the following three specific shoulder injection techniques: glenohumeral joint injection, acromioclavicular joint injection, and subacromial bursa injection. These are commonly used injection techniques for patients with shoulder pain [19]. To include all available videos, Python-based video data crawling of YouTube and hand searches of Google were conducted on 2 February 2022, using the following terms: “shoulder injection”, “glenohumeral joint injection”, “acromioclavicular joint injection”, and “subacromial bursa injection” (quotations included). These keywords were collaboratively chosen by the authors with reference to the results of Google trends. The videos included through Google searches were provided by video sharing platform such as Vimeo, medical associations, and medical device companies. Among the results of the Google searches, videos that were also searched on YouTube separately were excluded. All the retrieved videos were reviewed and evaluated. Commercials presented at the beginning and end of the videos were ignored.

**Figure 1:**
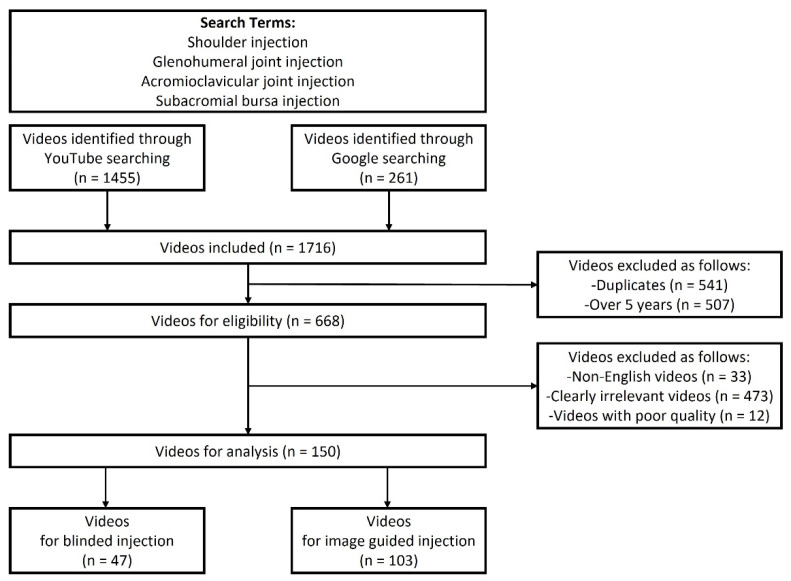
Flowchart for searching videos of shoulder injection treatments on YouTube and Google.

The exclusion criteria for videos were as follows: duplicate videos, videos uploaded before 1 January 2017, or over 5 years prior to the study, and non-English videos. Irrelevant videos (i.e., videos associated with other shoulder treatments, and veterinary videos) and very low-quality videos to score were also removed by authors’ judgments. The remaining videos were included for further analyses. A flowchart of the video selection process is shown in Figure 1.

The videos searched on YouTube and Google that were evaluated for this study were accessible to everyone. Ethics committee approval was not required because this study did not include human participants or animals.

### 2.2. Data Extraction and Categorical Distribution

On the day of the search, the basic descriptive characteristics of each video were collected. These included the title, uploader, and uniform resource locator, numbers of views, likes, and comments, duration (seconds), and posting days. Since November 2021, YouTube has not released the number of dislikes to ordinary viewers; therefore, we were unable to collect them. The number of views, likes, and comments of videos identified through Google searches, except for videos that were also searched on YouTube, were not collected owing to the lack of information. Based on the method described in a previous study, the viewing index (VI; views/posting days) was calculated whenever possible [20]. Subsequently, all videos were divided into the following two groups depending on whether an imaging instrument was used during injection treatment: the blinded group and the image-guided group. Separately, all videos were also categorized into two groups according to their type of uploader: the medical professional (an official university, professional organization/association, or physicians) and non-medical professional groups (an independent user, non-physician personnel, or unknown origin).

### 2.3. Scoring Systems

#### 2.3.1. mDISCERN for Reliability Assessment

The original DISCERN scale developed by Charnock et al. comprises 16 questions and assesses the reliability of written health information regarding treatments [21]. The mDISCERN scale used in this study was a simplified grading scale with only five questions designed to evaluate the videos (Table 1) [16,17].

This tool examines five aspects, including clarity, reliability, balance/bias, provision of information sources, and mention of uncertainty. Each of the five questions was scored on a two-point scale ranging from 0 to 1. The maximum potential score was 5, with significance in the reliability set at ≥3 [22].

#### 2.3.2. GQS for Overall Quality Assessment

The GQS developed by Bernart et al. was used to assess the overall quality of video content (Table 1) [18]. It is a five-point scale that assesses flow, ease of video use, and video quality. Scores of 1–2 points, 3 points, and 4–5 points were considered to indicate low-quality, moderate-quality, and high-quality, respectively [23].

#### 2.3.3. Shoulder Injection Score (SIS) for Educational Comprehensiveness Assessment

The SIS was developed by the authors of the current study to evaluate whether a beginner physician who has not previously performed shoulder injections, or who has less experience, can learn shoulder injection techniques through videos. The SIS contains the following 10 domains: indication, needle selection, injection materials, patient position, surface anatomy, sterilization, needle approach and insertion, injection target, contraindication and caution, and post-injection management (Table 1). Each domain was drafted by referring to articles analyzing videos of musculoskeletal injection therapy and to textbooks [24,25,26,27,28,29]. Subsequently, it was finally created based on discussions with four specialists in physical medicine and rehabilitation. If a domain was not described or shown in the video, it received 0 points for that item. Even if it contained at least one inaccurate explanation or action, it received 0 points. The SIS is based on the sum of points from the individual domains. The highest possible score for the video was 10 points, and the lowest score was 0. The higher the score, the more comprehensively accurate the information provided.

Two independent reviewers (H.E.C. and C.W.J.), who specialize in physical medicine and rehabilitation and have more than seven clinical years of shoulder injection treatments, evaluated each video using mDISCERN, GQS, and SIS after being trained to analyze the video in the same way. The content and information in each video’s footage were reviewed. If the video contained more than one shoulder injection technique, only the part of the test that was intended to be evaluated was assessed. Discrepancies in scores for the same video between reviewers were resolved by consensus until an agreement was reached.

### 2.4. Statistical Analysis

Descriptive data are presented as numbers (percentages) and means ± standard deviations. The Shapiro–Wilk test was applied to approximate the normality of data, and the Wilcoxon rank–sum test was performed to investigate the association between two groups. Pearson’s correlation coefficient was used to determine the relationship between mDISCERN and SIS, as well as GQS and SIS. Interrater reliability was measured separately for the scoring of the mDISCERN, GQS, and SIS using Cohen’s weighted kappa coefficient, with significance set at *p* > 0.6. All analyses were performed using the RStudio software (R version 4.1.2). Statistical significance was set at *p* value < 0.05 for parameters other than the interrater reliability.

## 3. Results

### 3.1. Basic Characteristics of Videos

A total of 1455 and 261 results were identified using YouTube and Google searches, respectively. After applying the exclusion criteria, 150 videos were selected and analyzed (Figure 1). The basic characteristics of the videos are summarized in Table 2.

The mean numbers of views and posting days were 6664.86 ± 26,270.10 and 808.30 ± 490.44 days, respectively. The mean VI was 7.57 ± 26.72. The mean numbers of likes and comments on the video were 56.37 ± 242.60 and 4.82 ± 24.38, respectively. The mean duration of the video was 167.92 ± 292.51 s. The average reliability (mDISCERN), overall quality (GQS), and educational comprehensiveness (SIS) scores of all videos were 2.01 ± 1.15, 1.88 ± 1.04, and 3.07 ± 2.91, respectively. The kappa scores indicated good agreement between the reviewers, showing that the interrater reliabilities for mDISCERN score, GQS, and SIS were 0.83, 0.85, and 0.83, respectively.

### 3.2. Reliability, Overall Quality, and Educational Comprehensiveness of Videos

**Figure 2:**
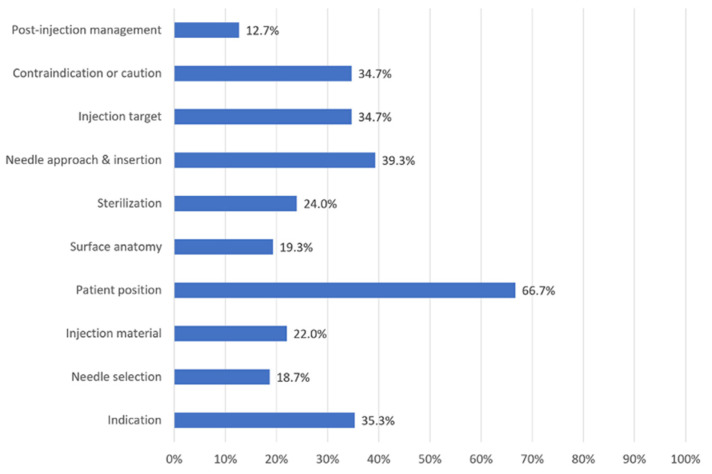
Percentage of videos scored in each of the 10 domains of the shoulder injection score.

Of the 150 videos selected, 49 (32.67%) contained highly reliable information (mDISCERN score: 5, N = 1; 4, N = 17; 3, N = 31; 2, N = 47; 1, N = 42; and 0, N = 12). Regarding the assessment of overall quality by the GQS, 41 (27.33%) videos were of moderate (N = 26, 17.33%), or high (N = 15, 10.00%) quality, which indicates that 109 (72.67%) videos were of low quality. Regarding the SIS, 114 (76.00%) videos scored no more than 5, with 77 of these (51.33%) scoring <3. Only 15 (10.00%) videos had a score of more than 7 (0, N = 37; 1, N = 31; 2, N = 9; 3, N = 11; 4, N = 15; 5, N = 11; 6, N = 14; 7, N = 7; 8, N = 6; 9, N = 7; and 10, N = 2). Except for the patient position (66.67%), the remaining domains have been fully explained in <40% of the included videos (Figure 2).

In particular, 19 (12.67%), 28 (18.67%), 33 (22%), 29 (19.33%), and 36 (24.00%) videos contained detailed information on post-injection management, information about needle selection, injection materials, explanation of surface anatomy, and proper sterilization, respectively. The mean scores of each SIS domain are summarized in Table 3.

The interrater reliability was significant for all scoring systems (0.87, 0.88, and 0.93 for mDISCERN, GQS, and SIS, respectively).

### 3.3. Categorical Analysis of Videos

#### 3.3.1. According to the Image-Guidance

Most videos (68.67%) covered the contents of the image-guided shoulder injection treatment, whereas the remainder covered the contents of the blinded shoulder injection treatment. When comparing the two groups, the Wilcoxon rank–sum test revealed significant differences in the mean VI (*p* = 0.03) and mean duration (*p* < 0.01).

The mean mDISCERN values for the blinded injection and image-guided injection groups were 2.38 ± 1.03 and 1.84 ± 1.17, respectively (*p* < 0.01). A significant difference was noted in the mean GQS, representing the overall quality of the video (2.47 ± 1.00 and 1.61 ± 0.94 for the blinded injection and image-guided injection groups, respectively; *p* < 0.006). The mean SIS of the blinded injection group (5.34 ± 2.55) was significantly higher than that of the image-guided injection group (2.01 ± 2.46) (*p* < 0.001). The mean scores for each domain are presented in Table 3. All domains showed significant differences between the two groups.

#### 3.3.2. According to the Uploader Type

According to the uploader type, a total of 128 videos (85%) were from the medical professional group, and 22 videos (15%) were from the non-medical professional group. Significant differences were found in the VI, views, and likes between the two groups.

Results of all three scoring systems were significantly higher in the videos of medical professionals than those of non-medical professionals (*p* = 0.001 for mDISCERN, *p* = 0.004 for GQS, and *p* = 0.001 for SIS). The mean scores for each domain of SIS are also presented in Table 3.

### 3.4. Correlation Analysis between Evaluation Tools

**Figure 3:**
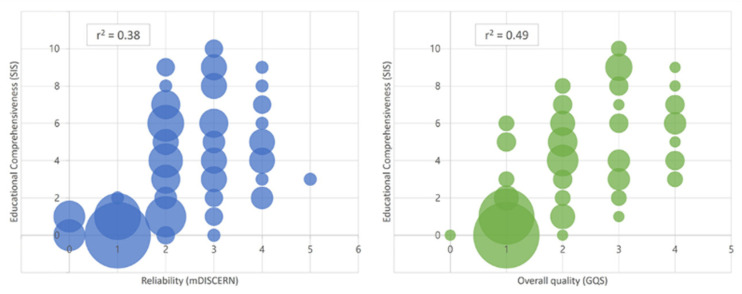
Relationship between the modified DISCERN, global quality score, and shoulder injection score.

**Figure 4:**
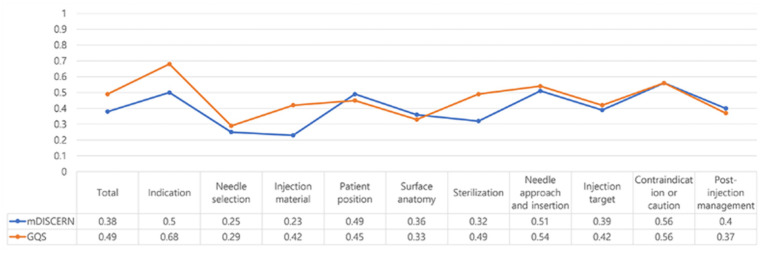
Correlation coefficients of the 10 shoulder injection score domains between the modified DISCERN and global quality score.

Figure 3 shows that there was a weak positive relationship between the mDISCERN score and SIS (r^2^ = 0.38). Moreover, a moderate positive relationship was observed between the GQS and SIS (r^2^ = 0.49). The correlation coefficient by domain is summarized in Figure 4.

Among the 10 domains, correlation coefficients of 0.5 or higher in both comparisons are indication, needle approach and insertion, and contraindications or cautions. Meanwhile, the domains that showed a weak positive correlation between the mDISCERN and SIS were needle selection, injection material, surface anatomy, sterilization, and injection target. Needle selection, surface anatomy, and post-injection management showed a weak positive correlation between the GQS and SIS.

## 4. Discussion

In this study, we assessed the reliability, overall quality, and educational comprehensiveness of all videos on shoulder injection treatments uploaded within 5 years. We found that only 32.7% of the videos contained highly reliable information, and 27.3% contained only 10% of high-quality videos. Only one video received five points in the mDISCERN, and no videos received five points in the GQS. Moreover, we found that the majority of videos on social media did not contain sufficient information to educate beginner physicians on shoulder injection techniques. Practical aspects, such as detailed information on which needle to choose, how to prepare the medicine for injections, and where to inject on the surface, were often neglected.

Several studies analyzed the videos of other clinical procedures on YouTube. In a study in which a total of 50 videos of epidural steroid injection were examined from the patient’s point of view, only 22% of the videos had highly reliable information, and only 34% were moderate to excellent in quality [25]. Similar findings were reported in other studies that investigated YouTube videos from the perspective of patient education in relation to transforaminal lumbar steroid injection and spinal injection [27,30]. The other two studies evaluated the suitability of YouTube videos regarding knee joint injection for educational purposes for medical professionals, and revealed that the videos cannot provide enough high-quality and reliable visual learning content for knee joint injection [24,26]. However, we do not believe that these results, including ours, mean that online videos are completely useless in the education of physicians for shoulder injections. We should not ignore the distinct characteristics of social media distributing videos other than traditional educational tools, such as worldwide influencing power, low price, and easy accessibility. Although online videos cannot replace traditional education in a real setting, some videos of very good educational comprehensiveness could be of great help to beginner physicians learning shoulder injections. In particular, it is expected to be useful in continuously reminding learned procedures or encountering new methods for techniques.

Interestingly, when broken down by the uploader type, the VI and likes of the medical professional group were significantly higher than those of the non-medical professional group. Although not statistically significant, the number of comments was higher in the medical professional group. This was consistent with the results of previous studies [20,31]. Considering that viewers, perhaps trainees and medical students, are most likely to find videos of specific injection procedures, this result may reflect their preference for videos made by medical professionals. Thus, the videos made by medical professionals had more engagement from viewers, as measured by VI, likes, and comments. Although videos in the medical professional group were superior in reliability, overall quality, and educational comprehensiveness compared to others, the overall scores were very poor. Therefore, when hospitals or physicians produce a video on shoulder injections, they should exert more effort to make videos with more practical and qualified information.

According to a review paper, pre-existing tools such as the mDISCERN and the GQS, which were not initially designed for video assessment, are still being used frequently [12,15]. The mDISCERN was developed to critically appraise users of consumer health information from the beginning, and the GQS is a grading system created to evaluate the overall quality of each website [17,18]. This implies that these assessment techniques might not be natural candidates to be evaluation tools of videos for medical personnel. Our results showed that the educational comprehensiveness of videos only has a weak positive relationship with reliability and a moderate positive relationship with the overall quality. In particular, in the results of the detailed analysis by domain, the practical parts, including needle selection, injection material, and surface anatomy, showed particularly low correlations between tools. In other words, educational comprehensiveness was not guaranteed even if the reliability assessed using the mDISCERN and overall quality using the GQS were high. In this regard, our study highlights the need for a new evaluation tool for injection videos for the educational purposes of physicians. Our findings are supported by the fact that numerous other assessors have actually developed a new evaluation tool [31]. This suggested the need for a new and universal evaluation tool suitable for evaluating videos for musculoskeletal injections.

One notable finding from our study is that videos of the image-guided injection group tended to have lower educational comprehensiveness than those of the blinded injection group. We suppose that this is probably because video providers assume that viewers who make an effort to search for those videos recognize basic information regarding shoulder injections. However, considering this, the videos are insensitive. Several videos about image-guided injection only focused on showing the procedure itself, without providing explanations. Basic injection sites, other than the description of image-guided injection, were often ignored. Furthermore, descriptions of the image, such as the anatomy of structures acquired in an image and how to obtain such images, were often neglected. Since image acquisition and description are essential for image-guided injection, the video cannot be considered educationally appropriate without at least explaining them. Providers of videos related to image-guided injection should make more efforts to create higher-quality videos.

Our study had an inherent limitation. The SIS was self-produced by the authors and did not receive peer review from other researchers. Although the authors have numerous musculoskeletal injection training experiences as experts who have been performing musculoskeletal injections for >7–20 years at university hospitals, more systematic and reliable injection evaluation tools need to be developed in the future. Another limitation is that we did not analyze all educational videos related to shoulder injection on the internet. Although we searched videos using four different keywords on two of the most popular platforms, Google and YouTube, there can be other educational platforms that may contain video materials that we could not include. Considering the dynamic nature of online platforms, many educational videos are produced and posted in real-time, and future studies including more videos are needed.

## 5. Conclusions

The majority of social media videos for shoulder injections, especially those about image-guided injections, were not suitable for educating beginner physicians due to their low educational comprehensiveness. Regardless of whether the videos are adequate for educational purposes, viewers prefer those produced by medical professionals. Therefore, medical professionals should pay more attention to uploading comprehensive high-quality videos for educational purposes. Meanwhile, the most used pre-existing tools, mDISCERN and GQS, were not enough to assess the educational comprehensiveness of social media videos. This allowed us to identify the need for a new scoring system to accurately evaluate musculoskeletal injection videos for education purposes. Future research needs to evaluate videos regarding other injections, and examine the development and demonstration of new evaluation tools.

## Figures and Tables

**Table 1:** Description of the components of the tools to evaluate videos with information on shoulder injection treatments.

Shoulder Injection Score (SIS) ^1^
1. Indication: Does it clearly explain the indications of injection procedure being performed?
2. Needle selection: Is the information about needle gauze selection clearly explained?
3. Injection material: Is sufficient information provided for injection materials? (Name and volume of the medicine used, and method of preparing the final substance to be injected)
4. Patient position: Is the patient’s posture shown or described?
5. Surface anatomy: Is the surface anatomy of the injected site clearly shown and described?
6. Sterilization: Does the procedure in the video strictly follow the principles of asepsis?
7. Needle approach and insertion: Are the instructions for depth, alignment, and direction movements of the needle included with the description of the three-dimensional structures?
8. Injection target: Does the video clearly describe what structure is targeted?
9. Contraindication or caution: Are the contraindications or cautions of the injection procedure being performed explained?
10. Post-injection management: Does the video explain further management plans for when patients can take a bath and/or exercise after the injection?
**Modified DISCERN (mDISCERN)** **^1^**
1. Clarity: Are the aims clear and achieved?
2. Reliability: Are valid sources cited (i.e., publication cited or speaker is board certified)?
3. Balance/bias: Is the information provided balanced and unbiased?
4. Provision of information sources: Are additional sources of information listed for patient reference?
5. Mention of uncertainty: Are any areas of uncertainty mentioned?
Global quality score (GQS)
1. Poor quality, poor flow of the video, most information missing, not at all useful
2. Generally poor quality and poor flow, some information listed but many important topics missing, of very limited use
3. Moderate quality, suboptimal flow, some important information is adequately discussed but some is poorly discussed, somewhat useful
4. Good quality and generally good flow, most of the relevant information is listed but some topics not covered, useful
5. Excellent quality and excellent flow, very useful

^1^ 1 point per question answered yes.

**Table 2:** Basic characteristics of the included videos.

**Variables, Mean (SD)**	**Total**	**Guidance**		***p*** **Value**
		**Blinded Injection**	**Image-Guided** **Injection**	
Videos, n (%)	150 (100)	47 (31)	103 (69)	N/A ^1^
VI ^2^	7.57 (26.72)	12.76 (39.32)	5.02 (17.29)	0.03
Views ^3^	6664.86 (26,270.10)	8564.37 (19,526.29)	5735.31 (29,057.19)	0.31
Posting days ^3^	808.30 (490.44)	758.30 (442.24)	832.77 (512.86)	0.49
Likes ^3^	56.37 (242.60)	96.43 (349.25)	36.77 (166.64)	0.12
Comments ^3^	4.82 (24.38)	8.15 (36.00)	3.29 (16.58)	0.08
Length	167.92 (292.51)	158.89 (108.07)	172.04 (345.93)	0.007
Reliability (mDISCERN ^4^)	2.01 (1.15)	2.38 (1.03)	1.84 (1.17)	0.003
Overall quality (GQS ^5^)	1.88 (1.04)	2.47 (1.00)	1.61 (0.94)	0.006
Educational comprehensiveness (SIS ^6^)	3.07 (2.91)	5.34 (2.55)	2.01 (2.46)	<0.001
**Variables, Mean (SD)**	**Total**	**Uploader Type**		***p*** **Value**
		**Medical** **Professionals** **^7^**	**Non-Medical** **Professionals** **^8^**	
Videos, n (%)	150 (100)	128 (85)	22 (15)	N/A ^1^
VI ^2^	7.57 (26.72)	8.82 (28.93)	0.85 (2.55)	<0.001
Views ^3^	6664.86 (26,270.10)	7845.63 (28,476.19)	331.63 (485.73)	0.012
Posting days ^3^	808.30 (490.44)	782.01 (498.36)	949.32 (428.49)	0.122
Likes ^3^	56.37 (242.60)	66.41 (263.20)	2.55 (4.89)	0.008
Comments ^3^	4.82 (24.38)	5.56 (26.21)	0.22 (0.55)	0.062
Length	167.92 (292.51)	180.18 (312.87)	96.59 (94.18)	0.098
Reliability (mDISCERN ^4^)	2.01 (1.15)	2.23 (1.04)	0.73 (0.94)	0.002
Overall quality (GQS ^5^)	1.88 (1.04)	1.98 (1.07)	1.27 (0.46)	0.004
Educational comprehensiveness (SIS ^6^)	3.07 (2.91)	3.40 (2.96)	1.18 (1.47)	0.001

^1^ Not applicable. ^2^ Viewing index (views/posting days.). ^3^ This figure was obtained only for YouTube videos. ^4^ Modified DISCERN. ^5^ Global quality score. ^6^ Shoulder injection score. ^7^ Medical professionals include official universities, professional organizations/associations, and physicians. ^8^ Non-medical professionals include independent users, nonphysician personnel, and unknown origin.

**Table 3:** Scores of included videos classified according to the image guidance and the uploader type for each domain of the shoulder injection score.

**Domains, Mean (SD)**	**Total**	**Guidance**		***p*** **Value**
		**Blinded Injection**	**Image-Guided Injection**	
Educational comprehensiveness (SIS ^1^)	3.07 (2.91)	5.34 (2.55)	2.01 (2.46)	<0.001
Indication	0.35 (0.48)	0.55 (0.50)	0.26 (0.44)	<0.001
Needle selection	0.19 (0.39)	0.34 (0.48)	0.12 (0.32)	0.001
Injection material	0.22 (0.42)	0.49 (0.51)	0.10 (0.30)	0.003
Patient position	0.67 (0.47)	1.00 (0.00)	0.51 (0.50)	<0.001
Surface anatomy	0.19 (0.40)	0.45 (0.50)	0.08 (0.27)	0.001
Sterilization	0.24 (0.43)	0.49 (0.51)	0.13 (0.33)	0.004
Needle approach and insertion	0.39 (0.49)	0.68 (0.47)	0.26 (0.44)	0.003
Injection target	0.35 (0.48)	0.51 (0.51)	0.27 (0.45)	0.005
Contraindication or caution	0.35 (0.48)	0.60 (0.50)	0.23 (0.42)	0.01
Post-injection management	0.13 (0.33)	0.23 (0.43)	0.08 (0.27)	0.008
**Domains, Mean (SD)**	**Total**	**Uploader Type**		***p*** **Value**
		**Medical Professionals** **^2^**	**Non-Medical Professionals** **^3^**	
Educational comprehensiveness (SIS ^1^)	3.07 (2.91)	3.40 (2.98)	1.18 (1.47)	0.001
Indication	0.35 (0.48)	0.40 (0.49)	0.09 (0.29)	0.006
Needle selection	0.19 (0.39)	0.20 (0.40)	0.09 (0.29)	0.215
Injection material	0.22 (0.42)	0.22 (0.42)	0.23 (0.43)	0.932
Patient position	0.67 (0.47)	0.70 (0.46)	0.45 (0.51)	0.023
Surface anatomy	0.19 (0.40)	0.21 (0.41)	0.09 (0.29)	0.191
Sterilization	0.24 (0.43)	0.27 (0.45)	0.05 (0.21)	0.021
Needle approach and insertion	0.39 (0.49)	0.45 (0.50)	0.05 (0.21)	<0.001
Injection target	0.35 (0.48)	0.41 (0.49)	0	<0.001
Contraindication or caution	0.35 (0.48)	0.39 (0.49)	0.09 (0.29)	0.007
Post-injection management	0.13 (0.33)	0.14 (0.35)	0.04 (0.21)	0.218

^1^ Shoulder injection score. ^2^ Medical professionals include official universities, professional organizations/associations, and physicians. ^3^ Non-medical professionals include independent users, non-physician personnel, and unknown origin.

## Data Availability

Data are available on request due to restrictions. The data presented in this study are available on request from the corresponding author.
